# A Rare Case of COVID-19 Vaccine-Induced Thrombotic Thrombocytopenia in a Young Patient

**DOI:** 10.7759/cureus.24355

**Published:** 2022-04-21

**Authors:** Osama Sobh, Najla AlSoofi, Afarah Alatifi, Lamees Alsulaim, Hassan Dahhan, Mohammed Abuselmiya, Ahmed AlJarallah, Marwa M Elmaghrabi

**Affiliations:** 1 Critical Care Medicine, King Saud Hospital, Qassim, SAU; 2 Rheumatology, King Saud Hospital, Qassim, SAU; 3 Critical Care, King Saud Hospital, Qassim, SAU; 4 Surgery, Unaizah College of Medicine and Medical Sciences, Qassim University, Qassim, SAU; 5 Neurology, King Saud Hospital, Qassim, SAU; 6 Internal Medicine, King Saud Hospital, Qassim, SAU; 7 Pulmonology, King Fahad Hospital, Qassim, SAU; 8 Microbiology, King Saud University, Riyadh, SAU; 9 Research, Center of Excellence for Research in Regenerative Medicine and its Applications (CERRMA) Alexandria University, Alexandria, SAU

**Keywords:** chadox1 ncov-19 vaccine, vitt, thrombosis, covid-19, astrazeneca

## Abstract

The syndrome of pulmonary SARS-Cov-2 resulted in significant morbidity and mortality, with new variants spreading rapidly. Vaccines to prevent COVID-19 have been developed to minimize the impact and severity; however, adverse effects of the vaccine have been documented in several studies. In our case, we report a case of a young female who presented to the emergency department with fever, dizziness, headache, vomiting, blurring of vision, numbness, and weakness of left upper and lower limbs. This weakness progressed rapidly to all limbs within two hours associated with altered behaviors and visual hallucinations. The family reported a history of the patient receiving her first dose of COVID-19 AstraZeneca vaccine 18 days before admission. Based on her clinical picture and investigation, she was diagnosed with vaccine-induced immune thrombotic thrombocytopenia (VITT). She was treated successfully with intravenous immunoglobulin (IVIG) and direct oral anticoagulant apixaban.

In a time when there is a strategic goal to vaccinate the global population from COVID-19 to inhibit the spread of infection and reduce hospitalization, this particular clinical scenario emphasizes the need for all clinicians to remain vigilant for rare complications of the COVID-19 vaccination.

## Introduction

Recently in late February 2021, a very rare prothrombotic disorder in combination with thrombocytopenia has been reported following vaccination with the adenovirus vector-based vaccines ChAdOx1 nCoV-19 from AstraZeneca/Oxford (marketed as Vaxzevria and Covishield) and recently Ad.26.COV2.S from Janssen (Johnson & Johnson) [1]. This novel disorder, known as vaccine-induced immune thrombotic thrombocytopenia (VITT) is associated with high titers of immunoglobulin G class antibodies directed against the cationic platelet chemokine, platelet factor 4 (PF4; CXCL4) [2]. These antibodies potently activate platelets via platelet FcγIIa receptors, with platelet activation greatly enhanced by PF4 [2,3].

VITT syndrome which mimics autoimmune heparin-induced thrombocytopenia (AHIT), has a distinctive feature of thrombosis in unusual sites including the splanchnic veins, adrenal veins, and cerebral and ophthalmic veins. Arterial thrombosis including ischemic stroke and peripheral arterial occlusion has also occurred, often in individuals with venous thrombosis. Females and those under 55 years of age are considered at higher risk [3,4].

VITT, with its high mortality and morbidity, should be suspected when a patient develops symptoms such as severe headache, blurred vision, vomiting, altered sensorium, focal neurological deficits, abdominal pain, leg pain, and/or swelling or dyspnoea, four to thirty days after vaccination [1,4]. Awareness of VITT, identification of early warning signs and symptoms, with proper management is crucial [4].

In this case study, we report a case of a young female with life-threatening cerebral vein thrombosis who showed good recovery when appropriate treatment of anticoagulation with a non-heparin direct oral anticoagulant and intravenous immunoglobulin (IVIG) was initiated.

## Case presentation

Our patient was a 23-year-old female who presented to the emergency room with fever, dizziness, headache, vomiting, blurring of vision, numbness, and weakness of the left upper and lower limbs. This weakness progressed rapidly to all limbs within two hours, associated with altered behaviors and visual hallucinations. The family reported that the patient received her first dose of COVID-19 AstraZeneca vaccine 18 days before admission. She was a known case of prolactinoma on cabergoline tablet 0.25 mg twice weekly.

On examinations, she was conscious and oriented but rather irritable, with heart rate 88/min, respiratory rate 20/minute, blood pressure 109/70 mmHg, temperature 38 degrees Celsius, oxygen saturation 96% on room air, a normal Glasgow coma scale score, and normal pupils. There was hypertonia in all limbs, power was 3/5 and 2/5 on the right and left upper and lower limbs, respectively. Reflexes were exaggerated and the plantar response was downgoing bilaterally. Cranial nerve and meningeal signs were grossly intact. While awaiting lab results, an initial brain computed tomography (CT) scan showed no major abnormality.

A post-vaccination meningoencephalitis possible diagnosis was made and management commenced accordingly including a small dose of dexamethasone 6 mg injection per day and a prophylactic dose of enoxaparin 40 mg s/c twice daily with close neuromonitoring in the intensive care unit (ICU).

**Figure 1:**
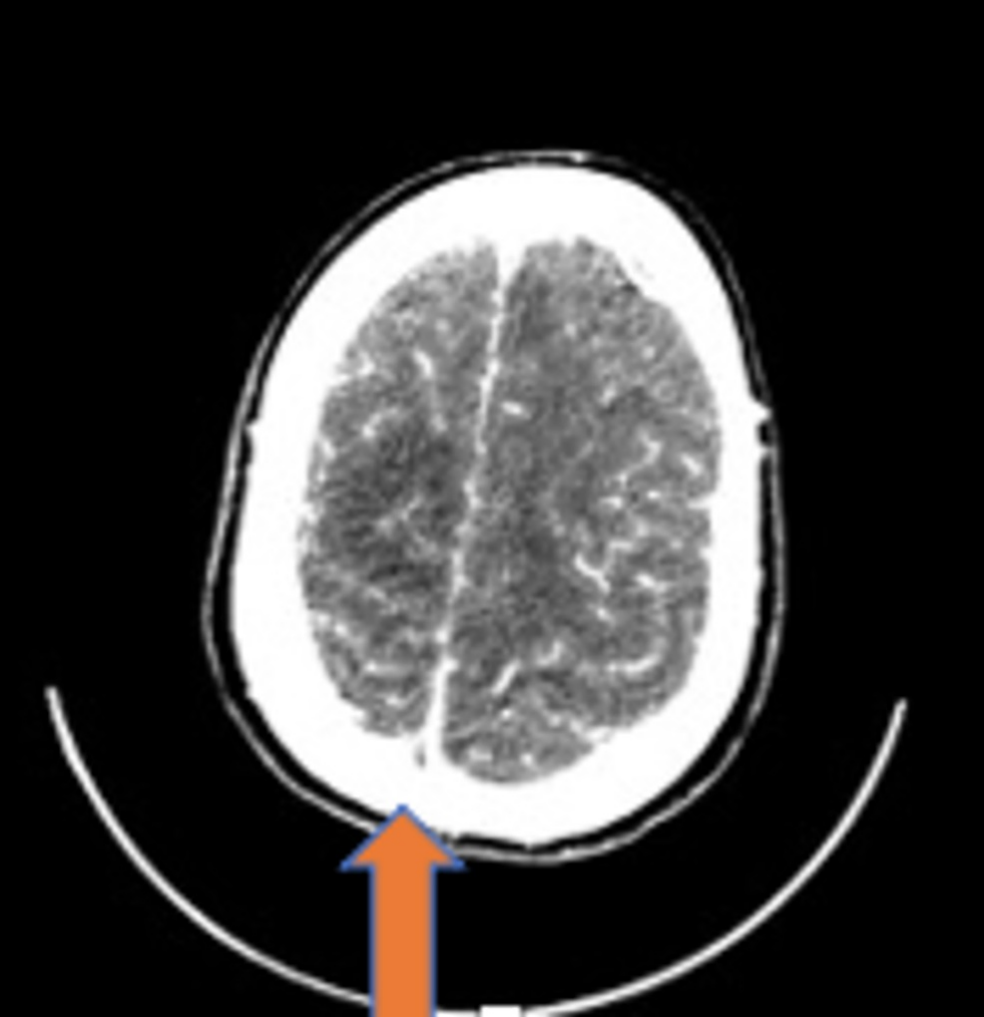
CT with contrast showing extensive superior sagittal sinus thrombosis

Labs results revealed pronounced thrombocytopenia; platelet count was 59 x 109/L, with extremely elevated d-dimer of 12600 ng/ml (normal range 70-500 ng/ml), fibrinogen level of 1.8 gm/l (normal range 1.50-4.00 g/l), and other normal pro-coagulant workups. A VITT diagnosis was established. A brain and cerebral sinuses venogram CT scan was urgently done that showed extensive superior sagittal sinus thrombosis with bilateral front parietal infarcts (Figure 1). Due to the high degree of suspension of VITT, an enzyme-linked immunosorbent assay (ELISA) test for platelet factor-4 antibody (anti PF4 antibody) was sent urgently to a higher center.

Based on the critical clinical picture of the patient, lab results, radiological findings, and without waiting for the results of the anti PD4 antibody, a multidisciplinary decision promptly started IVIG 1 g/kg once daily for two days. Enoxaparin was shifted to a non- heparin anticoagulant, direct oral anticoagulant (apixaban 5 mg twice per day) with close observation of the platelet level. As was expected, the anti PF4 antibody results were positive. A significant improvement in the patient neurological condition was obvious in a few days. Headache, blurring of vision, and weakness markedly improved. By day 10 of admission, the patient could walk freely without support. Her platelet counts return to normal.

## Discussion

With the rapid development and distribution of vaccines to control the COVID‐19 pandemic, trials showed reassuring safety signals, and many received approval [3,4]. However recently a rare, catastrophic hyper-coagulative syndrome associated with thrombocytopenia has been reported following vaccination with the adenovirus‐based vaccines. This syndrome has been designated as VITT [2].

The incidence of VITT following the AstraZeneca vaccine varies widely, most cases occurring in young adults (typically <60 years old), particularly young females, without overt risk factors or known thrombophilia [3,5]. Although the pathogenesis of VITT is uncertain, many laboratory findings support the theory that the mechanism of thrombocytopenia and thrombosis is similar to that of autoimmune HIT/T in that the anti-PF4 antibody is induced by polyanions in bacterial surface nucleic acids, including lipid A, instead of heparin. Some vaccine components have been suggested as essential aspects that could cause PF4 release and anti-PF4 antibody production in VITT, such as adenovirus DNA, spike protein, and/or neoantigen caused by the vaccine [3,6].

Most patients with VITT present with an unusual site of thrombosis, as the cerebral veins, splanchnic system, portal, and hepatic veins, pulmonary veins, cerebral artery, and other systemic sites have been reported [7,8]. Cerebral venous thrombosis incidence is higher, with neurological symptoms including headaches that are chronic and severe, vision problems, nausea, seizures, focal neurological deficits, multifocal symptoms, and changes in mental status. Consistent stomach pain, dyspnea, and/or leg pain edema are some of the other symptoms [4,7].

In our case, the young female with no history of previous thrombosis or family history of thrombophilia, presented with a new-onset of worsening headache, dizziness, and vomiting, and signs of increased intracranial pressure 18 days post ChAdOx1 nCov‐19 vaccination. Due to the significant mortality and morbidity associated with VITT, a variety of guidelines have been rapidly developed aiming at assisting physicians in diagnosing and managing this rare complication. Successful treatment requires a multidisciplinary approach, as in this case, which involved intensive care, hematology, neurology, and neurosurgery.

Any recently vaccinated patient with symptoms suggestive of VITT should have their platelet count, d-dimer, prothrombin time, partial thromboplastin time, and fibrinogen evaluated serially. Any abnormal results should be followed up with an ELISA anti-PF4 assay. A positive anti-PF4 test is significant, whereas a negative test in a patient with a high index of suspicion for VITT can be investigated further without delaying intervention [6].

Many guidelines urge commencing IVIG treatment immediately in any case where VITT is suspected. Thrombosis is a rare side effect of immunoglobulin, but it is still a required treatment to limit the progression of the disease [9].

A multicenter cohort study of cerebral venous thrombosis after vaccination against COVID-19 in the UK, by Perry et al., have shown that the consequences of VITT-associated cerebral venous thrombosis are worse than those of other forms of cerebral venous thrombosis. They have found that the proportion of patients with VITT who had died or were dependent on others for their care at the end of admission was significantly lower in those given IVIG and non-heparin anticoagulation in comparison to those who were not [10]. This supports our approach to our patients whose clinical picture, labs, and radiology findings were all highly suggestive of VITT. Though anti-PF4 antibody results were pending, immediate treatment with IVIG and direct oral anticoagulant apixaban, a non-heparin-based drug given, markedly accelerated the recovery of her general neurological symptoms and platelets count [9].

We compared our case with other cases that received the AstraZeneca vaccine which showed neurological symptoms and has shown that the duration after exposure was different, high suspect of VITT, and early intervention can improve the outcome (Table 1).

**Table 1:** Comparison of our case with the previous published VITT cases VITT: vaccine-induced immune thrombotic thrombocytopenia; IVIG: intravenous immunoglobulin.

Previous comparable VITT published cases				Our case
Age/ Gender	32Y/Female	26Y/Female	64Y/Male	23Y/Female
Clinical presentation	Headache, blurring of vision, giddiness and left hemiparesis.	Severe headache.	Lethargy malaise, and vague abdominal pain.	fever, dizziness, headache, vomiting, blurring of vision, numbness, and weakness of the left upper and lower limbs.
Received Vaccine	Covishield	AstraZeneca	AstraZeneca	AstraZeneca
Duration from vaccination to VITT	11 days	8 days	7 days	18 days
Platelets	120 × 10^9^/L	22 × 109/ L	20 x10^9 ^/L	59 x 10^9^/ L
d- dimer	1105 ng/ml	9452 ng/ml	36900 ng/ml	12600 ng/ml
Fibrinogen	2.6 mg/dl	173.8 mg/dl	400 mg/dl	180 mg/dl
Treatment	Mannitol, 3% saline, Intravenous levetiracetam, enoxaparin 40 mg twice daily subcutaneously, right parietal decompressive hemicraniectomy with evacuation of the intracranial hematoma, and IVIG.	IVIG, dexamethason, and Apixaban.	argatroban infusion, and IVIG.	Dexamethasone, Prophylactic dose of enoxaparin, IVIG, and apixaban.
Prognosis	Discharge after 17 days	Discharge after 6 days	Discharge after 14 days	Discharge after 10 days
Reference	Kotal et al. [1]	Khuhapinant et al. [11]	Al Rawahi et al. [12]	Our patient

## Conclusions

Regardless of the fact that COVID-19 vaccination may cause this unusual serious complication, experts recommended that the benefits exceed the risks. Our case report highlights the importance of increasing physicians’ awareness and early detection of warning of VITT signs after the COVID-19 vaccination in high probable cases. Prompt management with IVIG and non-heparin anticoagulation can significantly improve the patient’s outcome. As VITT has such a high mortality rate, treatment should start before the anti-PF4 antibodies ELISA tests confirm a positive result. To understand the underlying mechanism of VITT, more research cooperation is needed.
